# Brainstem Encephalitis Caused by *Listeria monocytogenes*

**DOI:** 10.3390/pathogens9090715

**Published:** 2020-08-30

**Authors:** Pengxu Wei, Ruixue Bao, Yubo Fan

**Affiliations:** 1 School of Biological Science and Medical Engineering and Beijing Advanced Innovation Center for Biomedical Engineering, Beihang University, 37# Xueyuan Road, Haidian District, Beijing 100083, China; pengxuwei@buaa.edu.cn; 2 Beijing Key Laboratory of Rehabilitation Technical Aids for Old-Age Disability, Key Laboratory of Intelligent Control and Rehabilitation Technology of the Ministry of Civil Affairs, National Research Center for Rehabilitation Technical Aids, No. 1 Ronghuazhong Road, Beijing Economic and Technological Development Zone, Beijing 100176, China; 3 School of Rehabilitation Medicine, China Rehabilitation Research Center, Capital Medical University, Beijing 100068, China; bacred@aliyun.com

**Keywords:** brainstem encephalitis, *Listeria monocytogenes*, MRI, vagus nerve

## Abstract

International outbreaks of listerial infections have become more frequent in recent years. *Listeria monocytogenes*, which usually contaminates food, can cause potentially fatal infections. Listerial cerebritis is a rare disease that is encountered mostly in immunocompromised or elderly patients. However, listerial brainstem encephalitis (mesenrhombencephalitis or rhombencephalitis) is found in persons who were formerly in good health, and recognizing this disease, particularly at its early stages, is challenging. Listerial brainstem encephalitis has high mortality, and serious sequelae are frequently reported in survivors. Early recognition and correct diagnosis, as well as the timely use of appropriate antibiotics, can reduce the severity of listerial infections. The trigeminal nerve is proposed as a pathway through which *L. monocytogenes* reaches the brainstem after entering damaged oropharyngeal mucosa or periodontal tissues. This review introduces the clinical manifestations, pathology, magnetic resonance imaging (MRI) findings, diagnosis, and treatment of listerial brainstem encephalitis. Moreover, it proposes that *L. monocytogenes* may also invade the brainstem along the vagus nerve after it infects enteric neurons in the walls of the gastrointestinal tract.

## 1. Introduction

*Listeria monocytogenes* is a Gram-positive bacterium that is common in our environment. It can be found in soil and animal/human excrement in addition to food and water. The bacterium is thought to live as a saprophyte that decomposes plant matter in its natural habitat. Tamed ruminants are likely important for preserving *L. monocytogenes* through constant stool-to-mouth transmissions [1]. Listerial infections caused by contaminated food, including meat, vegetables, edible aquatic life, and milk, are potentially fatal [2]. International outbreaks of listerial infections have increased in recent years. Listerial cerebritis is a rare disease and is encountered mainly in immunocompromised or elderly patients [3]. However, listerial brainstem encephalitis (mesenrhombencephalitis or rhombencephalitis) can be found in persons who were formerly in good health, and recognizing this disease, particularly at its early stages, is challenging. Listerial brainstem encephalitis has high mortality, and serious sequelae are frequently reported in survivors [4].

Early recognition and correct diagnosis, as well as the timely use of appropriate antibiotics, can reduce the severity of infection. This review focuses on the clinical manifestations, pathology, laboratory tests, and magnetic resonance imaging (MRI) findings [5] of brainstem encephalitis caused by *L. monocytogenes*.

The rhombencephalon consists of the pons, medulla, and cerebellum but does not include the midbrain [6]. Rhombencephalitis and brainstem encephalitis are slightly different anatomically, but these terms are used interchangeably by many authors [7]. In this review, we also use the two terms interchangeably. Notably, MRI and autopsy findings indicate that the brainstem and cerebellum in the same patient may be influenced (see Corresponding sections).

## 2. Epidemiology

International outbreaks of listerial infections, which is a rare disease, have become more frequent in recent years. The 2011 outbreak in the USA caused 147 cases of infection in 28 states and 33 deaths [8]. From January 2017 to July 2018, 1,060 cases of human listerial infections were reported in South Africa. This is the largest listeria outbreak in history [9]. From 2017 to 2018, a listeria outbreak occurred in Europe. Twelve infected people were identified in three countries with four dying because of or with the disease [10]. In the database of the Program for Monitoring Emerging Diseases, 126 listeria events were identified from 1996 to 2018. Three reports involved domestic animals or pets. Ninety-four events were human infections. Twenty-nine food recalls without clinical infections were attributed to contaminated materials [3].

Brainstem encephalitis occurs in up to 24% of patients with listeriosis [11]. Records collected in Norway from 1977 to 2000 indicated that brainstem encephalitis is common (19/172 in adult patients with listeriosis). However, brainstem encephalitis did not occur in 40 pregnant women with listeriosis. None of those 19 infected cases, however, were identified to have listerial brainstem encephalitis originally [12], which indicates the high under-recognition rate of this disease.

Listeria is commonly identified as an etiology of infectious rhombencephalitis [7]. From 1997 to 2010 in a hospital in the USA, 81 patients were found to have brainstem encephalitis. Four of these patients were classified as infected in accordance with etiologic categories with three probable cases of listeria and one case of central nervous system (CNS) aspergillosis [4]. From 1978 to 1988 in a Swiss hospital, 14 adult patients (aged 27–79 years) with acute *L. monocytogenes* rhombencephalitis were reported, among whom five died [13].

## 3. Clinical Presentation

Up to 29% of listerial infections may be asymptomatic [14]. Repeated sampling (approximately 8–9 times) detected *L. monocytogenes* in the feces of 60 out of 137 healthy pregnant women [15]. Listerial infection can also lead to abortion, stillbirth, or fetal death when occurring in pregnant women [16]. Listeriosis can manifest as febrile gastroenteritis [17,18], septicemia [19], and meningitis/meningoencephalitis [20,21]. CNS listeriosis is the frequently reported listerial infection (55% to 70% of cases) in nonpregnant adults [1].

From 1985 to 2014, 375 patients with listerial meningitis were diagnosed in the Netherlands with an overall high case fatality rate. Among 220 patients, 69 died several weeks after lumbar puncture for diagnostic purposes [20]. In people aged over 50 years, the clinical presentation of *L. monocytogenes* meningitis is distinct from that of bacterial meningitis caused by *Streptococcus pneumoniae* and includes high rates of extra-meningeal infection (72/109 vs. 1/22), respiratory failure within 48 h from admission (55/109 vs. 2/22), and Glasgow Coma Scale score <11 (77/109 vs. 21/22). In addition, in contrast to pneumococcal meningitis, *L. monocytogenes* meningitis is often detected in people in a coma who did not develop respiratory failure rapidly. Nevertheless, the symptoms and signs of *L. monocytogenes* meningitis lack specificity or sensitivity [2,22].

In 1921, *L. monocytogenes* was first isolated from a patient suffering from meningitis [11]. In 1957, brainstem encephalitis was first introduced as an infrequent type of listerial infection [23]. *L. monocytogenes* meningitis primarily occurs in patients with immunosuppression or those with coexisting diseases, the elderly, or neonates. By contrast, brainstem encephalitis predominantly occurs in previously healthy people. Many clinical manifestations, such as malaise, fever, headache, vomiting, and sweating, in the prodrome (4–10 days [11], or 5–15 days [13]) stage of brainstem encephalitis are not specific. Therefore, the early recognition of brainstem infection is challenging. After the prodrome stage, patients present progressive brainstem deficits, including cranial nerve palsy (facial paresis, diplopia, dysphagia, paretic soft palate, dysarthria, and paresthesias in the trigeminal region), and cerebellar dysfunction/ataxia. The motor and/or sensory deficits of extremities (long-tract signs, e.g., hemiparesis- or tetraparesis, spasticity, or increased tendon reflexes), respiratory distress, consciousness impairment (from confusion to coma), seizure, fever, and meningitis, may also be present [11,13,24,25,26,27,28,29,30]. In 123 cases with listerial rhombencephalitis, the most frequently reported symptom is an eye movement deficit, which is followed by headache, an altered mental status, limb ataxia, nausea/vomiting, nuchal rigidity, dysarthria, dizziness/vertigo, and dysphagia. Several possible causes of cranial nerve signs have been suggested: invasion of a cranial nerve and then its nucleus, intra-axonal spreading from a region in the brainstem to other regions, and a space-occupying effect originating from abscesses and accompanying edema in the brainstem [31].

## 4. Anatomy and Physiology of the Brainstem

The brainstem, which is a small and compact structure in the human CNS, bridges the cerebral hemispheres, cerebellum, and spinal cord [32], and participates in maintaining basic vital functions, such as the regulation of the heart rate, breathing, sleeping, eating/swallowing, walking, and vestibular, somatic, and visceral senses [33].

**Figure 1:**
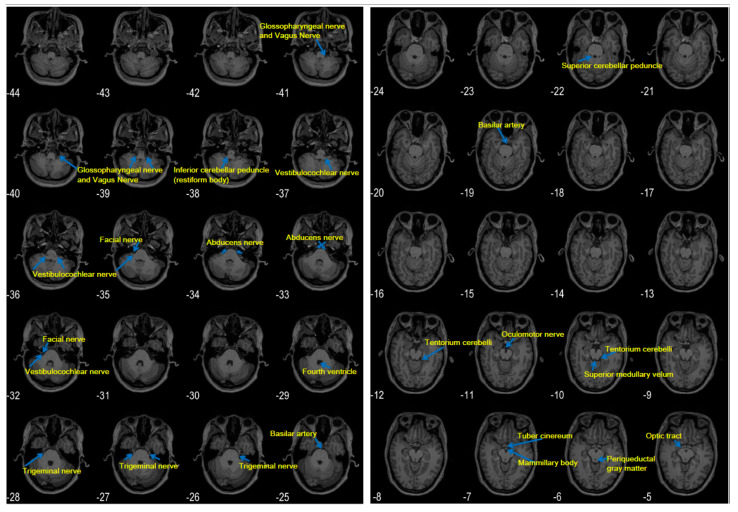
Cranial nerves in high-resolution magnetic resonance (MR) images. Images were produced by using the statistical parametric mapping (SPM) toolbox. High spatial-resolution T1 images (0.5 mm × 0.5 mm in the axial plane, 1 mm slice thickness) were collected with a 3T magnetic resonance imaging (MRI) scanner. Numbers indicate locations in the z-axis. Note the different structural features between (z=) “−37,” showing the root of the vestibulocochlear nerve, and (z=) “−39,” showing the roots of the glossopharyngeal/vagus nerves. In these high-resolution images, the root of the glossopharyngeal and vagus nerves cannot be visually separated. Structures are indicated on the basis of References [34,35].

The roots of cranial nerves III–XII exit from the brainstem. However, only some cranial nerves can be seen in clinical MR images [34,35]. Additional detailed structures of the brainstem and cranial nerves on MRI may be presented with a high spatial resolution (Figure 1). Notably, among cranial nerves III–XII, the trigeminal nerve is almost conspicuous in an MRI. The vestibulocochlear and facial nerves are also easily seen. The roots of the glossopharyngeal and vagus nerves are jacent and difficult to differentiate. The roots of the oculomotor and abducens nerves are thin but often visible. The roots of the trochlear and hypoglossal nerves are very thin and may not be seen.

**Figure 2:**
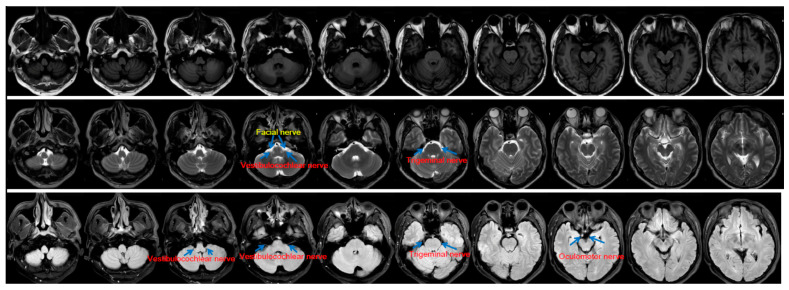
Cranial nerves on clinical routine MRI. T1, T2, and Flair MRI images with a low spatial resolution (5-mm slice thickness in the axial plane with a 1-mm gap between slices) acquired by using a 3T MRI scanner are shown in the first, second, and third rows. Note that fewer cranial nerves can be seen in this figure than in Figure 1.

The vestibulocochlear, glossopharyngeal, and vagus nerves depart from the lower pons and the medulla on the lateral sides in a cephalic–caudal order (Figure 1). Clinical routine MRI has low spatial resolution (e.g., as shown in Figure 2). Thus, thin nerve roots, such as the hypoglossal nerve, are difficult to see. Nevertheless, thick cranial nerves, i.e., the trigeminal, vestibulocochlear, and facial nerves, are easily found. Recently, a review that included 123 cases of *L. monocytogenes* rhombencephalitis concluded that abnormal findings for the hypoglossal nerve are reported only occasionally, although the cranial nerves (fifth, seventh, ninth, tenth, and twelfth) supplying the oropharynx are commonly affected [31]. The introduced phenomena (i.e., the trigeminal nerve is thick and almost conspicuous, the glossopharyngeal and vagus nerves are jacent and difficult to differentiate, and the roots of the trochlear and hypoglossal nerves are too thin to be seen) may account for numerous reports involving the trigeminal nerve and the scarcity of MRI reports involving the vagus nerve.

The dorsal vagus motor nucleus contains parasympathetic neurons that send axons via the vagus nerve to the ganglia in the gastrointestinal wall and in other abdominal organs. The motor nucleus acts as an important component that modulates visceral motor function (e.g., the activity of gastrointestinal smooth muscles) and secretion (e.g., stomach acid) [36,37].

The medulla oblongata has four vagal nuclei. The three main nuclei are the dorsal vagus motor nucleus, ambiguous nucleus, and solitary nucleus. The fourth is the spinal trigeminal nucleus, which receives major inputs from the trigeminal nerve but has minor input from the vagus nerve [38]. The spinal trigeminal nucleus relays the sensory inputs from the ipsilateral face to the contralateral thalamus. The spinal trigeminal nucleus incorporates sensory information from all three branches of the trigeminal nerve in addition to the facial, glossopharyngeal, and vagus nerves [39]. Extensive interconnections are found among the spinal nucleus of the trigeminal nerve and the solitary nucleus, ambiguous nucleus, and dorsal motor nucleus [40]. A functional MRI study demonstrated that the stimulation of the sensory auricular branch of the vagus nerve evokes extensive activities in brainstem regions, including the solitary nucleus, spinal trigeminal nucleus, and other areas [41]. Thus, a pathogen may be transmitted along the vagus nerve to the spinal trigeminal nucleus or the vagal nuclei.

Patients with brainstem lesions may present dysphagia or dysarthria [31]. Except for the stylopharyngeus, which is innervated by the glossopharyngeal nerve, the pharyngeal muscles are innervated from the pharyngeal plexus by the vagal pharyngeal branch [37]. Only solitary nuclei receive visceral afferents through the facial, glossopharyngeal, and vagus nerves and innervate the salivatory, hypoglossal, dorsal vagal motor, and ambiguous nuclei. Patients with dysphagia or dysarthria may present an abnormal gag reflex. The glossopharyngeal nerve is the afferent pathway of this reflex, whereas the efferent pathway comprises the glossopharyngeal and vagus nerves originating from the ambiguous nucleus [35]. The central pattern generator of deglutition is considered to be in the region of the solitary nucleus [42,43]. Electrical stimulation with ring electrodes to the pharynx can promote the recovery of dysphagia in patients with a stroke [44]. This effect indicates that the sensory input from the pharynx can improve the swallowing function by modulating the brainstem nuclei.

The brainstem contains cholinergic, dopaminergic, noradrenergic, and serotonergic nuclei with projections from/to other areas [33,45]. The cuneiform nucleus (CN) and the pedunculopontine nucleus (PPN) in the midbrain participate in gait control [46,47]. The scopes of the CN and PPN are defined in a histochemically-based atlas [48]. Lesions in these regions may result in gait disturbance [49]. The periaqueductal gray matter (PAG) has been regarded as a node that links the cerebellum and participates in motor control [50,51,52]. In reported cases of listerial brainstem encephalitis, MRI demonstrated lesion enhancement in the PAG and right CN/PPN in T2 images but only in the right CN/PPN in T1 images [4]. Another study found MRI signal hyperintensities in the scopes of PAG, CN, and PPN in patients with listerial brainstem encephalitis [29].

## 5. Pathology

To date, the genus *Listeria* consists of 17 species, among which *L. monocytogenes* and *Listeria ivanovii* are considered pathogens [53]. Mice and rats are not natural hosts of *L. monocytogenes*, whereas humans and ruminants are natural hosts of this bacterium [11]. The histopathological characteristics of the listerial rhombencephalitis lesions of ruminants are analogous to those of humans, which indicates similar pathogenesis in both hosts [54]. In northern Italy, 60% of the 20 identified cases of ruminant listerial brainstem encephalitis were found to be related to listeriosis outbreaks in humans, which indicates that ruminants may be the natural hosts of *L. monocytogenes* strains that cause listeriosis epidemics among humans [55].

The gastrointestinal tract is considered the primary site through which *L. monocytogenes* invades the human body. Food contamination by low levels of this bacterium is associated with human cases of *L. monocytogenes* infections [56], possibly due to the multiplication of *L. monocytogenes* in refrigerators, the virulence of the bacterial strain, and the susceptibility of the host [1]. *L. monocytogenes* is an opportunistic pathogen. The susceptibility of the host is important in terms of whether the host becomes asymptomatic or presents the disease after exposure to *L. monocytogenes*. Most patients have pathological defects (risk factors) that affect T-cell-mediated immunity. Ingested *L. monocytogenes* invades intestinal tissues after surviving in gastric acid. The spread of the bacterium across enterocytes may lead to enteritis. The blood or lymph conveys the bacterium mostly to the liver. Some *L. monocytogenes* also arrive in the spleen and lymph nodes in the mesentery. *L. monocytogenes* in the liver can be eliminated if the immune response is adequate. In patients with depressed/compromised immunity, unlimited proliferated bacteria in the liver may enter circulation. The bacterium is thought to invade the brain via the blood or by migration along the cranial nerves [1].

A study that reviewed 123 cases of listerial rhombencephalitis reported that, among 96 patients with data for the evaluation of immunocompetence, 69.8% were identified as immunocompetent [31].

Efforts to isolate the bacterium from the cerebral spinal fluid (CSF) of ruminants with encephalitis always fail, which suggests that, during infection, *L. monocytogenes* rarely enters the CSF [57]. In addition, brain autopsy findings support the hypothesis that the bacterium enters the brainstem via the cranial nerves [30]. Thus, hematogenous infection is unlikely to lead to listerial brainstem encephalitis [11]. In mice, inoculating *L. monocytogenes* in a cranial nerve or facial muscle can induce clinical brainstem encephalitis with histological changes [58]. Autopsy findings in nine human cases with listerial brainstem encephalitis revealed inflammatory infiltrates primarily in the nuclei of the cranial nerves innervating the oropharynx, which indicates that the pathogen invades the brainstem along the cranial nerves [30]. Two patients with brainstem encephalitis and culture-identified listeriosis bacteremia presented sensory trigeminal nerve dysfunction (paraesthesia in the face) prior to any other neurological symptoms. Additionally, MRI detected contrast-enhancing lesions in the sensory trigeminal tract in the brainstem [31]. Collectively, *L. monocytogenes* may infect local nerve endings when injuries exist in the mucosa of the oropharynx and cavum nasi and then reach the brainstem via axonal migration [11]. A route via the conjunctiva [59] may also be possible.

In sheep that are cutting, changing, and losing teeth, the inoculation of *L. monocytogenes* into the endodontium follows a route from the infected trigeminal nerve at the dental terminals to the brain via an ascending neuritis [60]. Notably, dental diseases are highly prevalent across the world and pose a serious public health challenge [61], but are often under-reported [62]. Therefore, we suggest that the frequently reported involvement of the trigeminal nerve in listerial rhombencephalitis [31] may partly stem from damaged dental and periodontal defense mechanisms due to dental diseases, which provide a route for the food-borne *L. monocytogenes* to invade the brainstem through the trigeminal nerve. An autopsy study of more than 200 natural cases of listerial encephalitis in cattle, sheep, or goats found that, except for the frequent involvement of the trigeminal nerve, lesions are also detected in solitary nuclei and oculomotor and facial nerves. These findings suggested that *L. monocytogenes* may enter the brainstem via axonal migration along other nerves [54].

**Figure 3:**
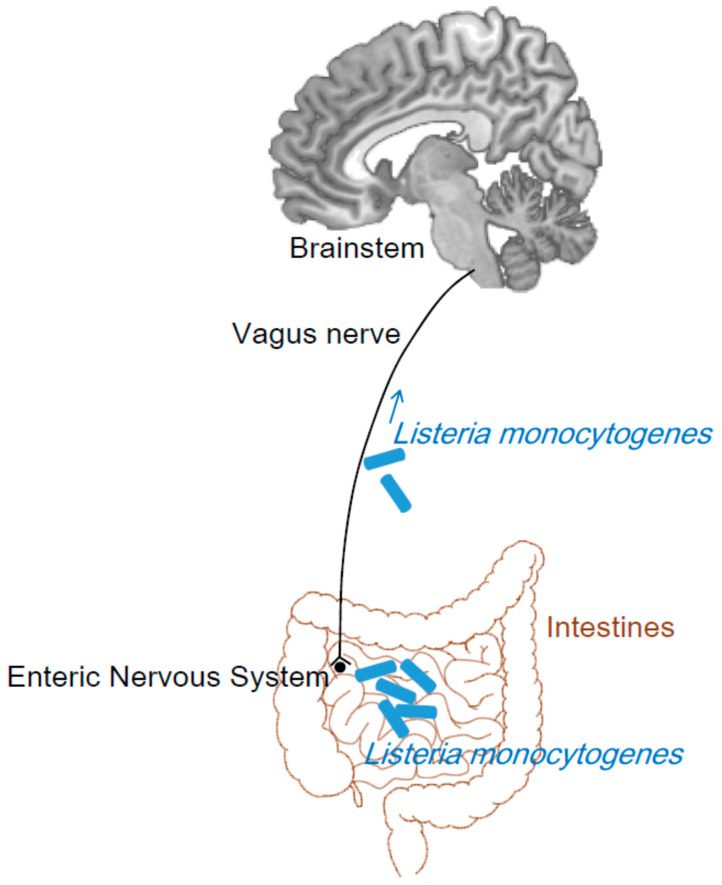
Vagus nerve as a possible route for *L. monocytogenes* to invade the brainstem. The dorsal vagus motor nucleus sends axons via the vagus nerve to the ganglia in the gastrointestinal wall. After ingested *L. monocytogenes* invades intestinal tissues, these axons may be a route for *L. monocytogenes* to intrude into the brainstem. The brain is generated from an anatomical template in the SPM Anatomy toolbox.

In humans, rhombencephalitis caused by *L. monocytogenes* often present gastrointestinal symptoms, such as nausea and vomiting [11]. Two of the three reported cases with *L. monocytogenes* rhombencephalitis presented a history of gastroenteritis (one month for one patient and no definite duration for the other patient) prior to the involvement of the cranial nerves [31]. Nausea and vomiting are frequently seen in another group of 14 adult patients with *L. monocytogenes* rhombencephalitis [13]. This phenomenon is difficult to attribute to the infection of the brainstem through intra-axonal transport along the trigeminal nerve or other cranial nerves. Therefore, we propose that the vagus nerve connecting the gastrointestinal tract and brainstem may be a route of the intra-axonal transport of *L. monocytogenes* (Figure 3).

The long distance of the vagus nerve can account for the delay (approximately 4 weeks) between gastrointestinal tract infection and brainstem involvement in certain cases. The slow transition of an enteric pathogen to the brain through the vagal nerve is proposed as the cause of Parkinson′s disease [63]. In a mouse model, the vagus nerve in the small intestine or colon infected by a modified rabies virus transports the virus into the nucleus tractus solitarius of the brainstem [64], which proves the existence of such a pathway. Prior to listerial brainstem encephalitis, during the prodrome (4–10 days [11], or 5–15 days [13]) stage that presents nausea, vomiting, fever, and sweating, the bacterium may be transported along the vagus nerve. After arriving in the brainstem, it can spread from one part to other parts of the brainstem. The mechanisms by which *L. monocytogenes* intrudes into the brainstem through the trigeminal nerve also permit the entry of the bacterium in the brainstem along the vagus nerve.

The brainstem vagal nuclei, e.g., the solitary tract nucleus and nucleus ambiguous, are found to be involved in listerial brainstem encephalitis in animals [54] and humans [30], which indicates the possibility of these nuclei as targets of the transported bacterium. This review is specifically written to introduce the vagus nerve as the route for *L. monocytogenes* to invade the brainstem.

Notably, the trigeminal nerve is thicker than the vagus nerve and other cranial nerves on brainstem MRI (Figure 1). Hence, the hyperintensities of the trigeminal nerve are more frequently observed and reported in magnetic resonance (MR) images than those of other cranial nerves. The application of high-field MRI and high spatial resolution may be important for detecting the hyperintensities of the vagus nerve.

*L. monocytogenes* is recently found to be highly heterogeneous. By contrast, hypervirulent clones likely cause diseases, particularly CNS and maternal–neonatal listeriosis [53]. This result indicates that hypervirulent strains may be prone to causing CNS listeriosis, such as brainstem encephalitis, in previously healthy people without evident risk factors.

The autopsy findings of one of two patients who died due to listerial brainstem rhombencephalitis revealed the presence of a diffuse purulent exudate over the brainstem, cerebellum, and hemispheric convexities, particularly the choroid plexus and ependyma of the lateral and IV ventricles, with the exudate covering denuded ependyma from the ventricular wall. Subependymal perivascular and parenchymal leucocytic infiltration was also found. The other patients showed discrete subarachnoid hemorrhage around the brainstem but no purulent exudate. Multiple abscesses were found in the medulla, pons, and midbrain. Numerous areas of suppurative encephalitis confined to the brainstem were observed. The rupture of abscesses into the ventricles led to ventriculitis and diffused subependymitis [13]. A more recent study including nine cases in Norway showed that listerial brainstem encephalitis primarily involved the medulla oblongata. In addition, leucocytic infiltration occurred primarily in one nucleus/tract of the oropharynx-innervating cranial nerve, i.e., the fifth, seventh, ninth, tenth, and twelfth cranial nerves. Inflammation was found primarily in the brainstem and occasionally in other brain regions, including the putamen, thalamus, hippocampus, temporal lobe, cerebellum, and meninges. The inflammatory lesions consisted of bacteria, leukocytes, and occasional micro-abscesses [30].

## 6. Radiographic Features

MRI is superior to computed tomography (CT) for examining abnormalities in a patient suffering from brainstem diseases, such as rhombencephalitis [27]. CT revealed brainstem widening, abscesses in the brainstem, and cerebellum, hemorrhagic lesions in the vermis, and hydrocephalus in 14 cases of *L. monocytogenes* rhombencephalitis. However, CT failed to detect any lesions in five out of 13 patients [13].

In 123 patients with *L. monocytogenes* rhombencephalitis, MRI localized bacterial abscesses in the medulla oblongata, cerebellum, pons, midbrain, and supratentorial regions. In a patient on the 12th day after the appearance of symptoms, such as headaches, MRI using a 1.5 T scanner revealed contrast enhancement but failed to identify the corresponding structure. MRI on the 16th day showed the abnormality at the left trigeminal nerve root. Moreover, the principal sensory nucleus and spinal nucleus were also involved. The MRI of the other two patients (one at an earlier stage, another at a similar stage) also revealed abnormal trigeminal nerve root signals [31].

MRI disclosed T2 hyperintense signals in the dorsal part of the pons in a female patient with listerial rhombencephalitis without gadolinium-enhanced lesions. MRI also revealed white matter involvement, e.g., in the frontoparietal subcortical region [27]. Another report presented two cases with listerial rhombencephalitis with one case demonstrating ring contrast enhancement on T1 images while the other shows T2 lesions in the brainstem and enhancement in other brain regions [29]. In another report, gadolinium-enhanced MRI revealed a T1-contrast-enhanced lesion of the right trigeminal nerve in addition to the hypoglossal nucleus, solitary tract, and spinal trigeminal nucleus on the right side [30]. In other studies, MRI also showed contrast-enhanced lesions (minor abscesses) in the brainstem [28,29].

Gadolinium-containing magnetic resonance contrast agent can enter the brain tissue only if the blood–brain barrier has been damaged. Gadolinium enhancement may not be observed or show only mild contrast in the early stage of encephalitis. During a brain abscess, the abscess wall shows contrast enhancement [65,66,67].

## 7. Diagnosis, Treatment, and Prognosis

*L. monocytogenes* is a Gram-positive bacterium. However, CSF examination may not reveal *L. monocytogenes*. However, pleocytosis, polymorphonuclear leucocytes, and increased protein concentration can be observed [13,29]. Positive rods were identified by Gram staining in only one patient in a group of 13 patients. However, 10 of these patients had positive CSF cultures (blood cultures positive in six cases and negative in four cases) [13].

In addition to infections, the etiology of rhombencephalitis includes autoimmune diseases and paraneoplastic syndromes [7]. Differential diagnosis needs to be considered for viral infection or inflammatory demyelinating CNS diseases, e.g., multiple sclerosis, isolated brainstem syndrome, or neuromyelitis optica [2,67].

Considering the various mechanisms by which listerial brainstem encephalitis may cause cranial nerve signs and the various forms of symptoms/signs of brainstem involvement [30,31], the suspicion of listerial brainstem encephalitis should also immediately arise even in the absence of fever or neck stiffness in patients with undetermined meningitis or other forms of listeriosis upon the appearance of brainstem damage symptoms/signs [13]. In the default of specific or sensitive clinical manifestations, a tentative diagnosis of listerial brainstem encephalitis can be performed when the clinical background indicates the possibility of brainstem involvement, and/or when the patients show a history of possible *L. monocytogenes* infection, including fever and the consumption of contaminated food.

Brainstem encephalitis caused by *L. monocytogenes* results in an overall mortality of up to 51% [26]. Hence, early suspicion and treatment with appropriate antimicrobial therapy remain the best option to reduce the high death rate and sequela severity of this disease [27,28]. Treatments should be started empirically with suitable antibiotics as early as possible, and the delayed initiation of antimicrobial therapy should be avoided. Intravenous ampicillin (likely combined with one of the aminoglycosides) or penicillin has been proven to be effective for the treatment of listerial meningitis and brainstem encephalitis, with the former often considered as the first choice. Vancomycin, meropenem, or linezolid may also be used for subjects who are allergic to ampicillin/penicillin [7,27,29,31,68,69]. Although mortality is 32.2% [31], and 55% of survivors have neurologic sequelae [7], effective intervention in the early stage may lead to full recovery within several weeks [29].

## 8. Conclusions

The trigeminal nerve (facial and cranial nerves III, IV, and VI) has been proposed to be the route through which *L. monocytogenes* reaches the brainstem after entering damaged oropharyngeal mucosa or periodontal tissues. In this scenario, we propose that *L. monocytogenes* may also intrude into the brainstem along the vagus nerve after it infects enteric neurons in the walls of the gastrointestinal tract. We also present high-resolution and clinical MRI to illustrate the visibility of different cranial nerves to show that the lesions of thick cranial nerves, such as the trigeminal nerve, are more likely to be found than those of thin cranial nerves (e.g., cranial nerves IV and XII). The roots of the glossopharyngeal and vagus nerves may not be seen in clinical MRI and are difficult to differentiate even by high-resolution MRI. This difficulty may account for the low rate of positive findings for thin and vagus nerves on MRI examination. High-resolution MRI may have greater probability of detecting abnormalities of the vagus nerve. However, the vagus nerve biopsy [70] should be the gold standard to determine whether a viral etiology and the intra-axonal transport [64,71] exist. Determining the exact route through which the bacterium invades the brainstem may help prevent listerial brainstem encephalitis.
